# Supplementary material for: Mitigating the impact of motor impairment on self-administered digital tests: a longitudinal cohort study in stroke
Source: eClinicalMedicine. 2025 Sep 2;88:103469. doi: 10.1016/j.eclinm.2025.103469 (PMC12433494; doi:10.1016/j.eclinm.2025.103469)
Supplement: Supplementary Figures and Tables [file mmc1.docx]

Supplementary Methods

MRI data acquisition and pre-processing

Structural MRI data, including high-resolution 1mm^3^ isotropic T1-weighted and T2-FLAIR (Fluid Attenuated Inversion Recovery) sequences were acquired using a Siemens 3T scanner at three and twelve months after stroke. T1-weighted sequences were obtained using the following parameters: repetition time (TR)=2300ms, echo time (TE)=2.98ms, flip angle (FA)=9^o^, field of view (FOV)=265x265mm, 160 slices. T2-FLAIR acquisition parameters were: TR=5000ms, TE=395ms, FA=120^o^, FOV=250x250mm, 160 slices.

Lesion masks and white matter hyperintensities (WMH) masks were manually delineated on the T2-FLAIR images guided by the clinical diffusion weighted imaging (DWI) obtained acutely after the stroke. All masks were created by trained research teams and checked by experienced neurologist (FG). Non-brain voxels were removed from T1 and T2-FLAIR images using the Brain Extraction Tool (BET) from FMRIB’s Software Library (FSL), using robust brain centre estimation and a fractional intensity threshold of 0.5. T2-FLAIR brain images, along with lesion and WMH masks, were registered to T1 space with 6 degrees of freedom (DOF), using FMRIB’s Linear Image Registration Tool (FLIRT). Subsequently, T1 brain images and masks were registered to the standard Montreal Neurological Institute (MNI) 1mm brain template using 12 DOF and trilinear interpolation. An inverted lesion mask was included in the registration to down-weight the influence of the lesion in the registration and thus minimize distortions associated with the registration of lesioned tissue. Volumetric measures of the standard-space lesion and WMH masks were calculated for each subject using FSL. The lesion overlap maps are presented in Supplementary Figure 9, separately for stroke lesion and white matter hyperintensities.

Supplementary Results

**Supplementary Figure 1**: **Difficulty scale assigned to each of the 18 task.** Y axis shows scaled difficulty of each trial type in arbitrary units derived from the computational model. X axis shows the trial types per task. **Trail-Making Task.** The task includes 14 “Circles” and “Stars” trials that do not require switching, and 14 “Switching” trials that require participants to alternate between categories. It is observed that, generally, the “Switching” trials are more challenging than the “Circles-only” and “Stars-only” trials. The first trial is typically the most difficult, regardless of whether switching is required. **SRT (Simple Reaction Time) and Auditory Attention Tasks.** Those tasks are modelled using the inter-stimulus interval (ISI) between the current and previous trial. Trials with consistent ISI are generally easier with faster and more accurate responses. In contrast, variable ISI leads to slower responses and increased errors. **Rule Learning Task.** The task consists of eight sequential “rule changes” (labelled 1 to 8) that participants must figure out. The rules become progressively more challenging, with the hardest rules presented towards the end of the task. **Motor Control Task.** Difficulty is modelled based on the distance (in pixels) between the ‘current’ target and the previously seen target. Larger distances require more complex hand movements, leading to slower response times


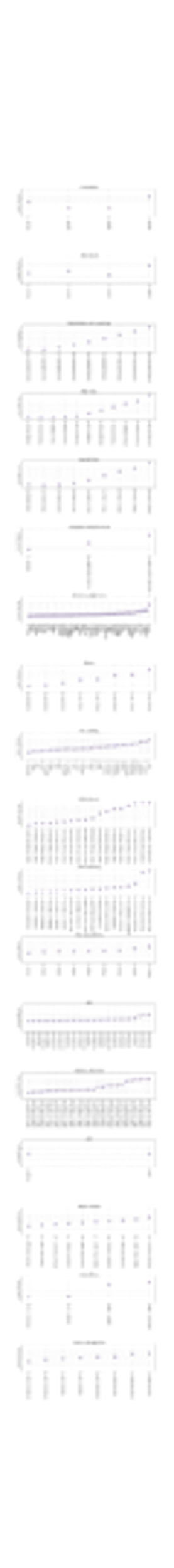


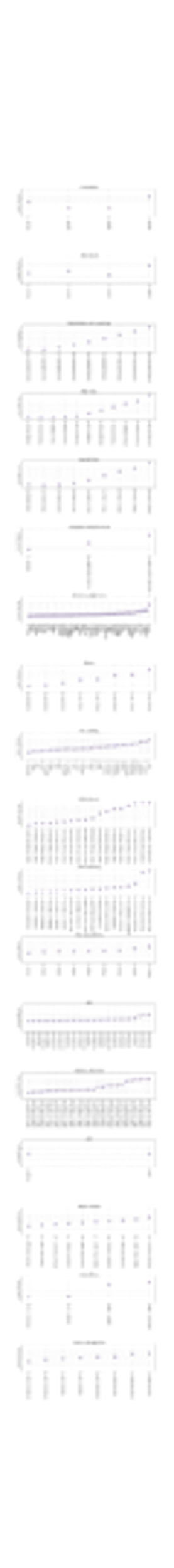


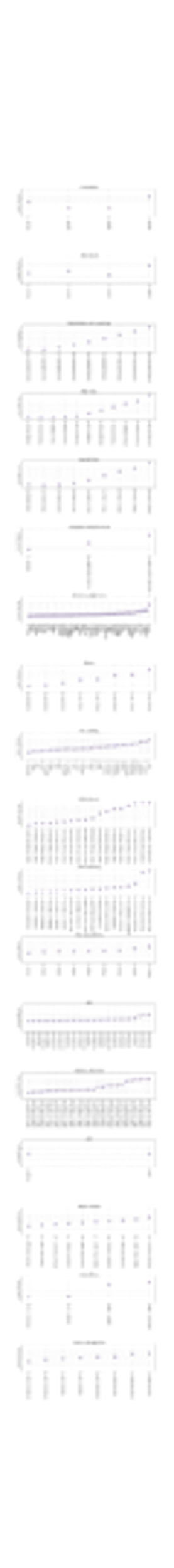


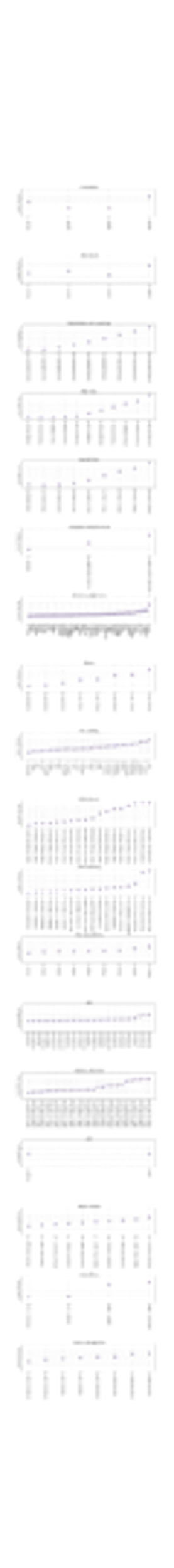


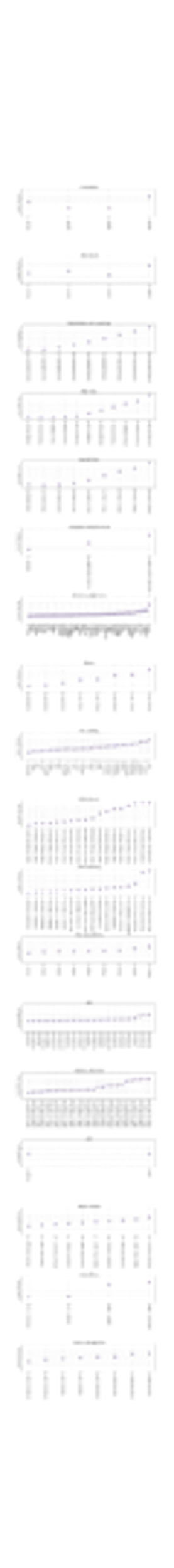


**Supplementary Figure 2** - Outcome measure distributions for each task. Yellow bars refer to standard raw metrics derived for each task (Standard Accuracy and Median Reaction Time in milliseconds). Purple refers to modelled performance measures (Cognitive Index and Response Delay Time in standard deviation units).

Across all 18 tasks, raw accuracy metrics were predominantly observed to follow a negatively skewed gamma distribution, expected for such clinical research cohort where the patients tend to suffer from mild-moderate impairment levels.  In contrast, the modelled Cognitive Index more closely approximated to a Gaussian distribution, thereby mitigating ceiling effects, and showing its ability to capture more variability in cognition. Speed-related measures (e.g., median reaction time and Response Delay Time) typically followed a gamma distribution with a slight positive skew. This is in keeping with speed-based measures capturing a wider dynamic range.


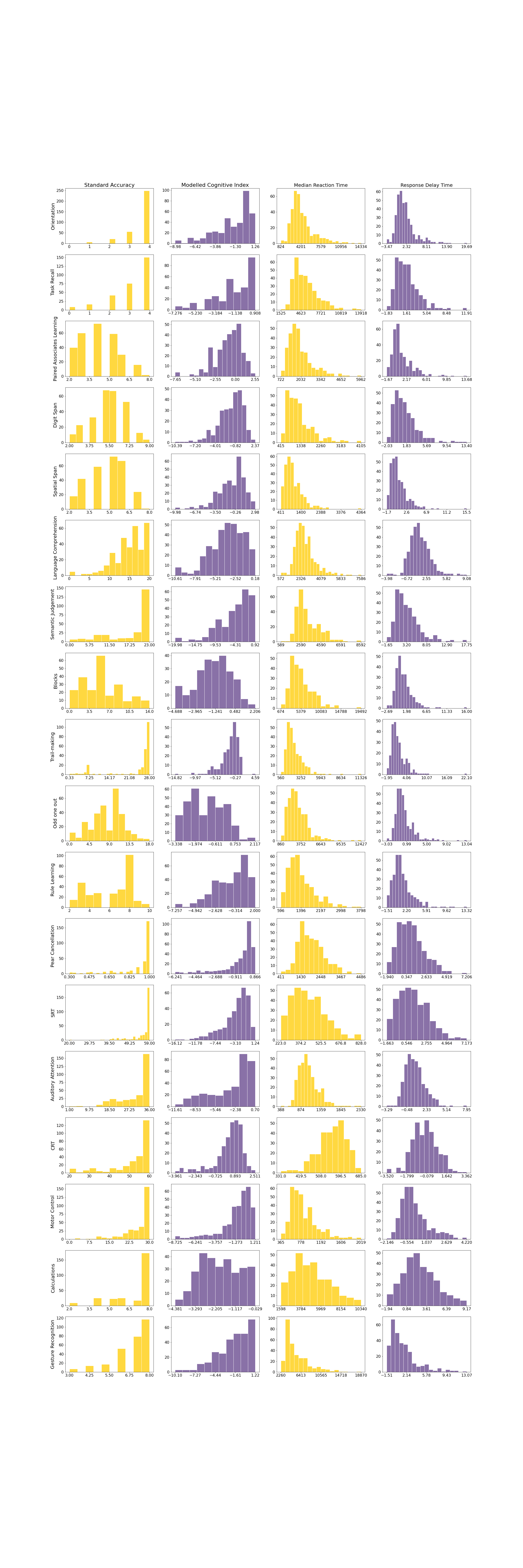


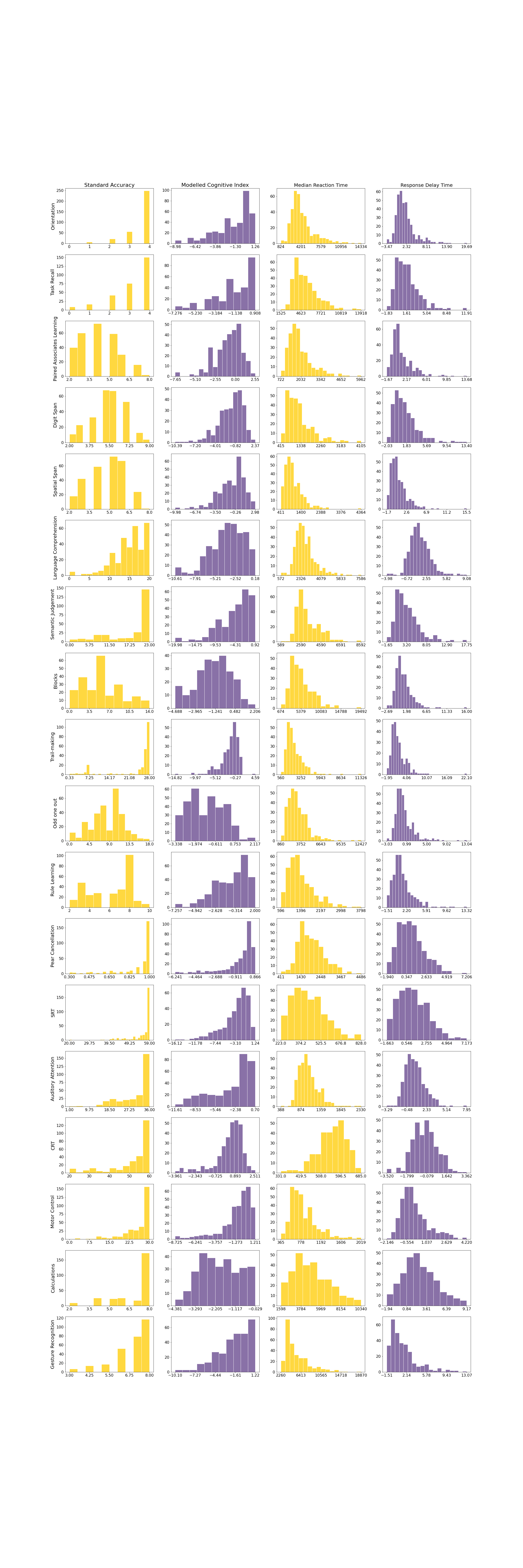


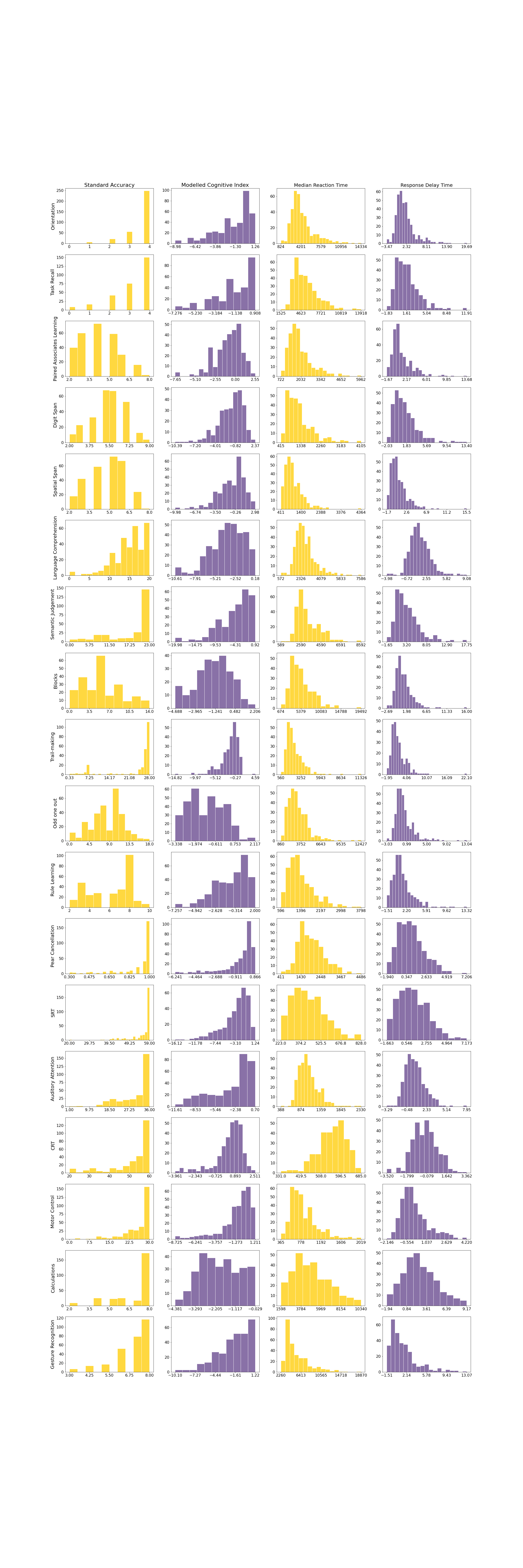


**Supplementary Figure 3** – Graphical summary of the IDoCT framework. IDoCT provides measures of cognitive ability (i.e. the Cognitive Index), response delay time (DT), and trial-difficulty (D) by parsing time-courses comprising trial-by-trial accuracy, reaction time and trials’ descriptive labels from a population sample.


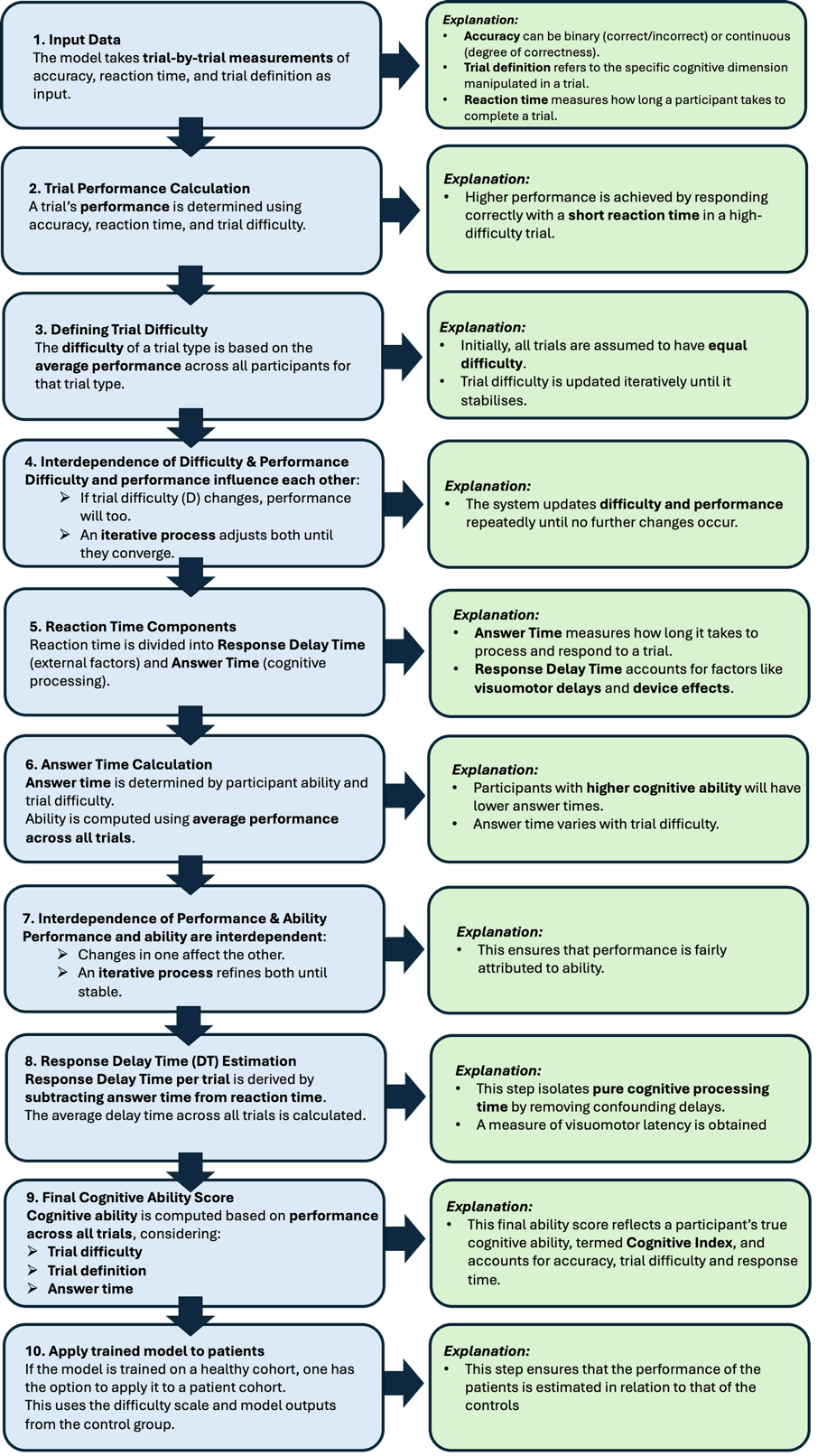


**Supplementary Figure 4** – Relationship between cognitive scores and demographic and clinical variables. The modelled Cognitive Index is displayed in purple. Standard accuracy scores are shown in yellow. The strength of the association is shown in standard deviation units, along with 95% confidence intervals. The reference value for education refers to patients that were at most educated to Secondary school (≤16 years old, GCSE level).


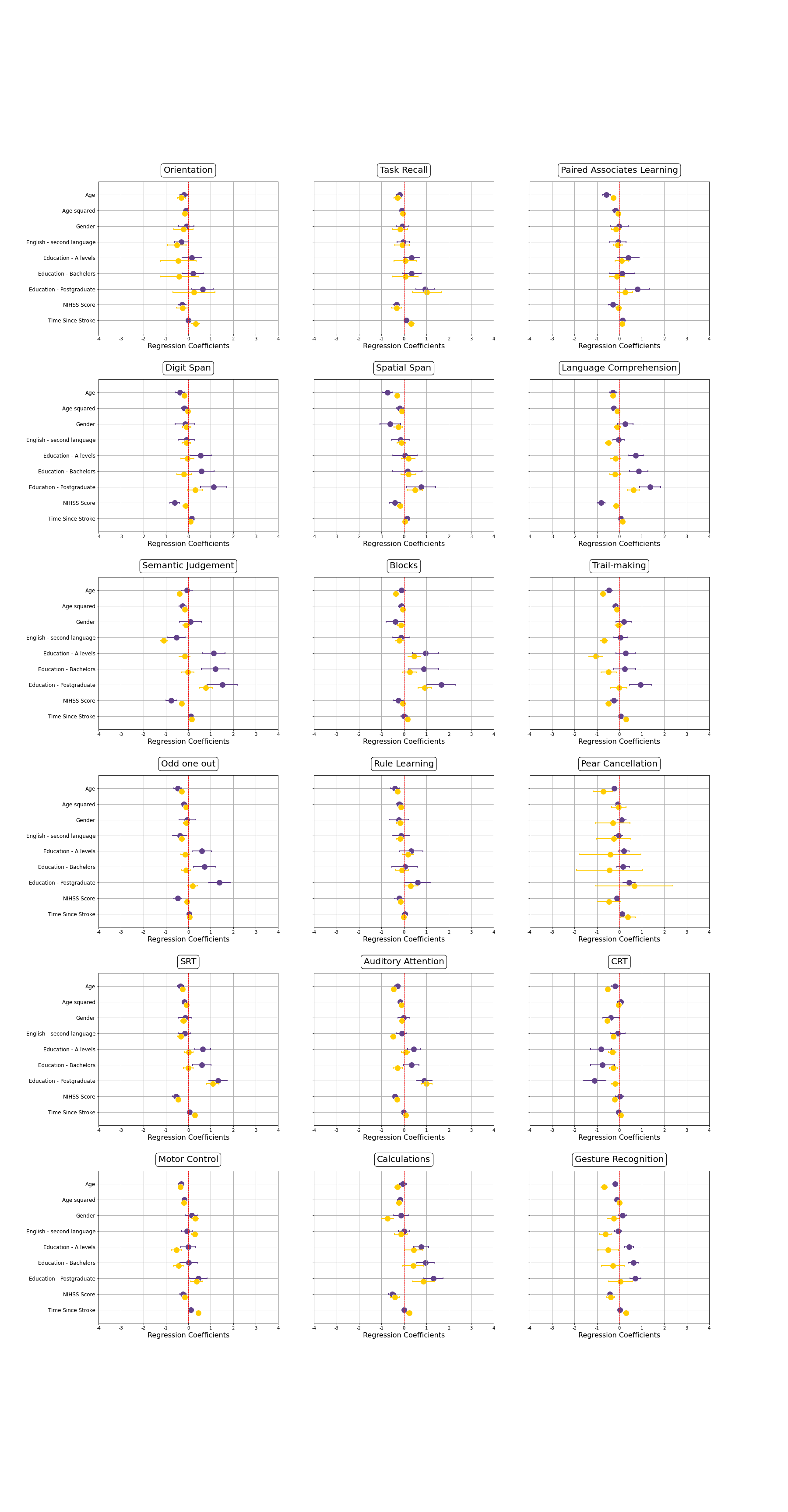


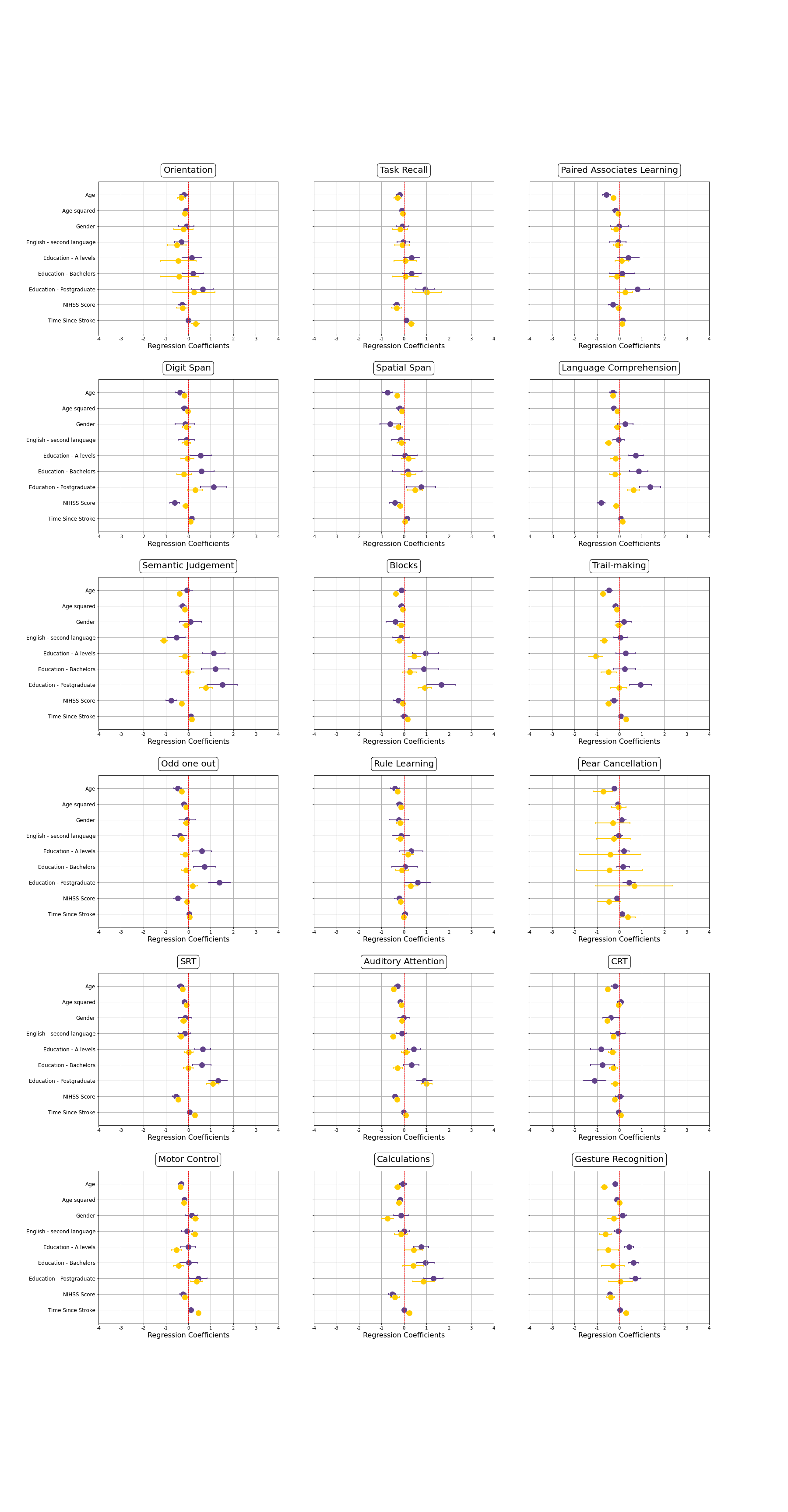


**Supplementary Figure 5** – The effect of using an impaired hand when completing the self-administered cognitive tasks. The absolute strength of the effect is shown in standardised beta coefficients. Modelled metrics are shown in purple (Cognitive Index= solid purple; Response Delay Time= hatched purple), and standard metrics are shown in amber (Standard accuracy= solid amber; median reaction time in milliseconds (hatched amber). Asterisk denotes significant effect of hand-motor impairment after FDR correction for the four performance metrics within each task. Significant effects indicate worse/slower performance when using an impaired hand.


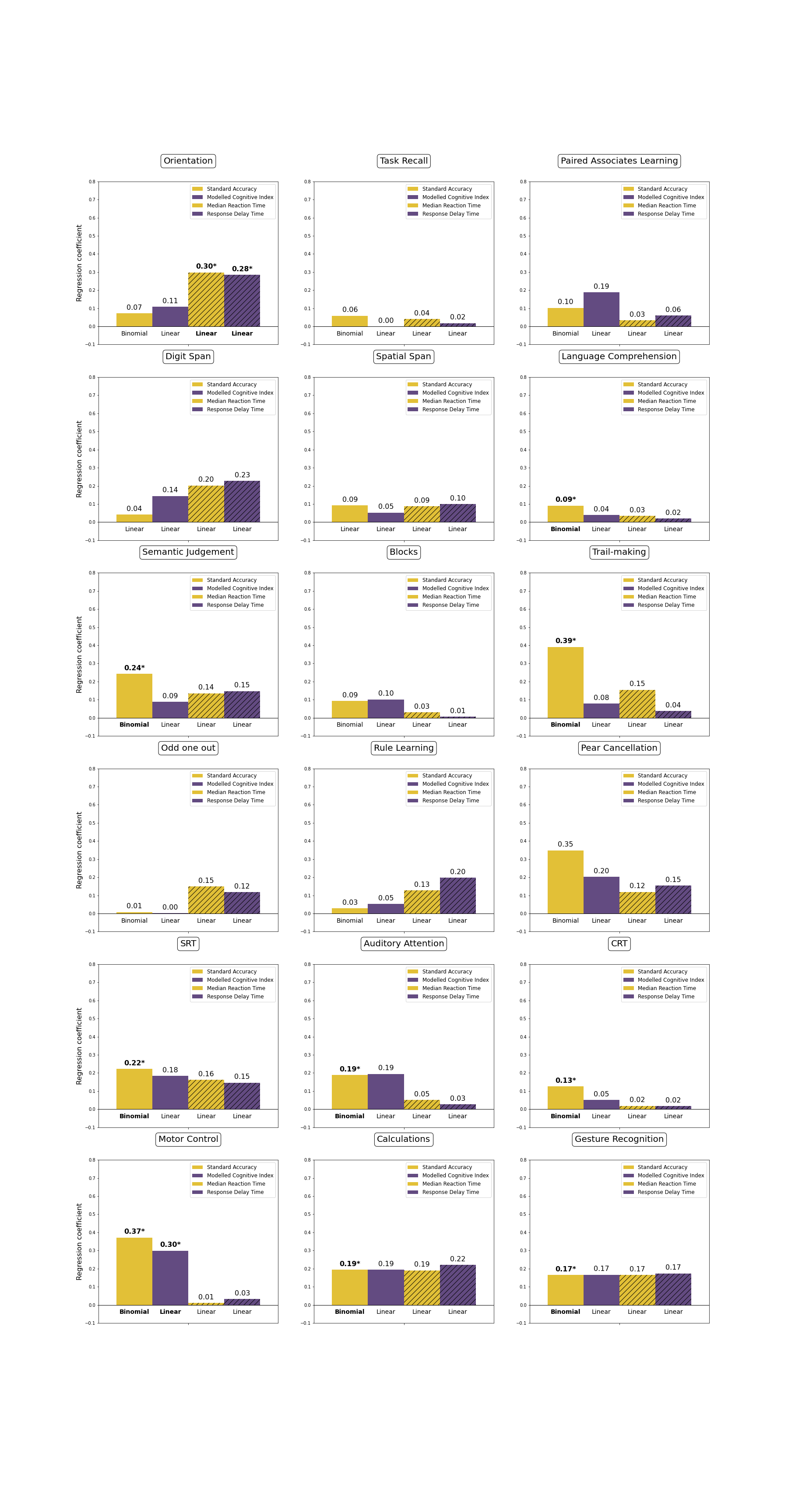


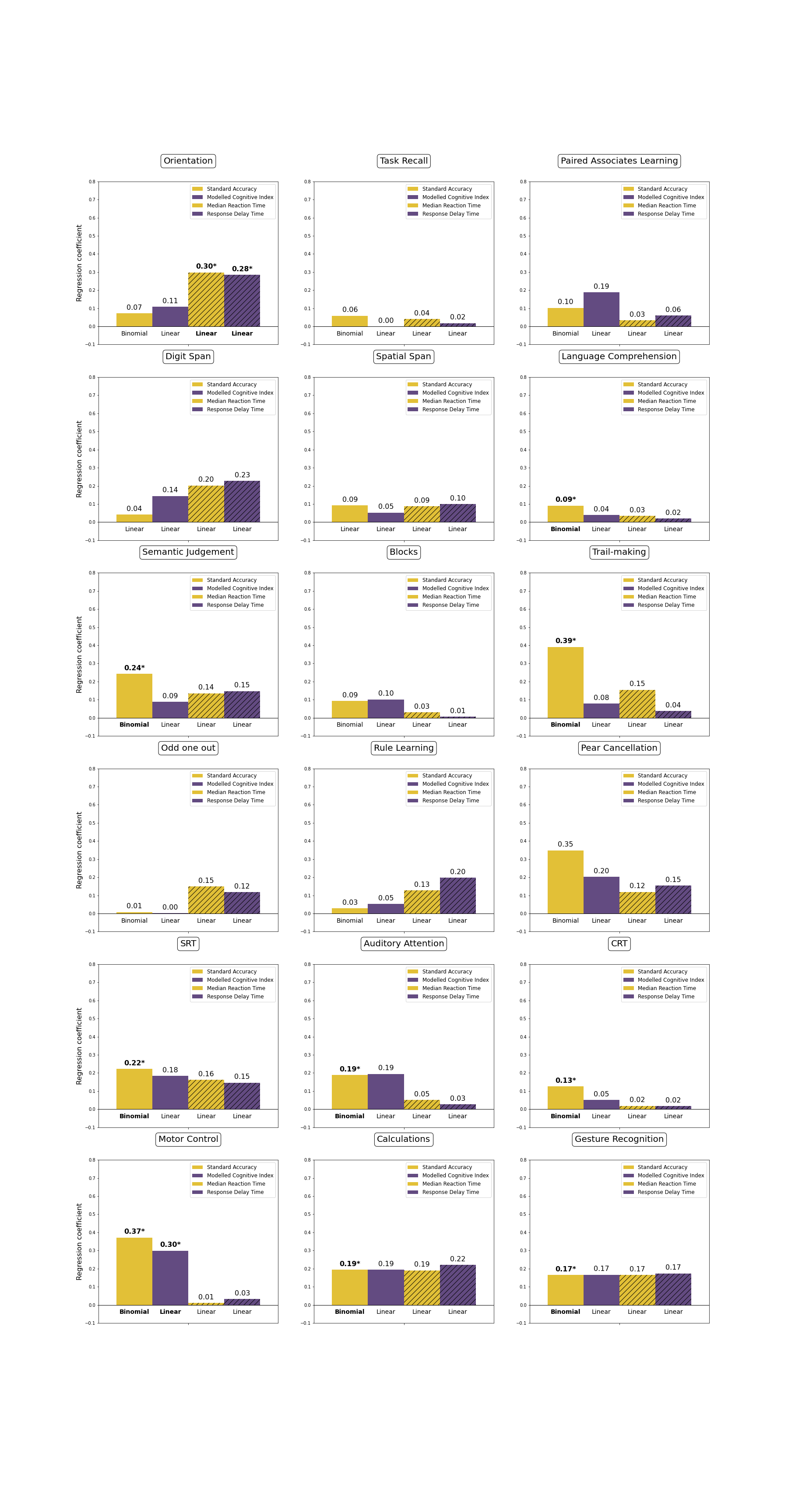


**Supplementary Figure 6** – Global cognitive performance estimated via PCA analysis is related to functional outcomes post-stroke (Instrumental Activities of Daily Living- IADL). **Top panel:** Correlation between global standard accuracy metrics (G_standard accuracy_) and functional abilities across the acute, sub-acute and chronic phases of the stroke. **Bottom panel:** Correlation between the global modelled Cognitive Index (G_cognitive Index_) and IADL scores across the acute, sub-acute and chronic phases of the stroke. P-values are uncorrected.


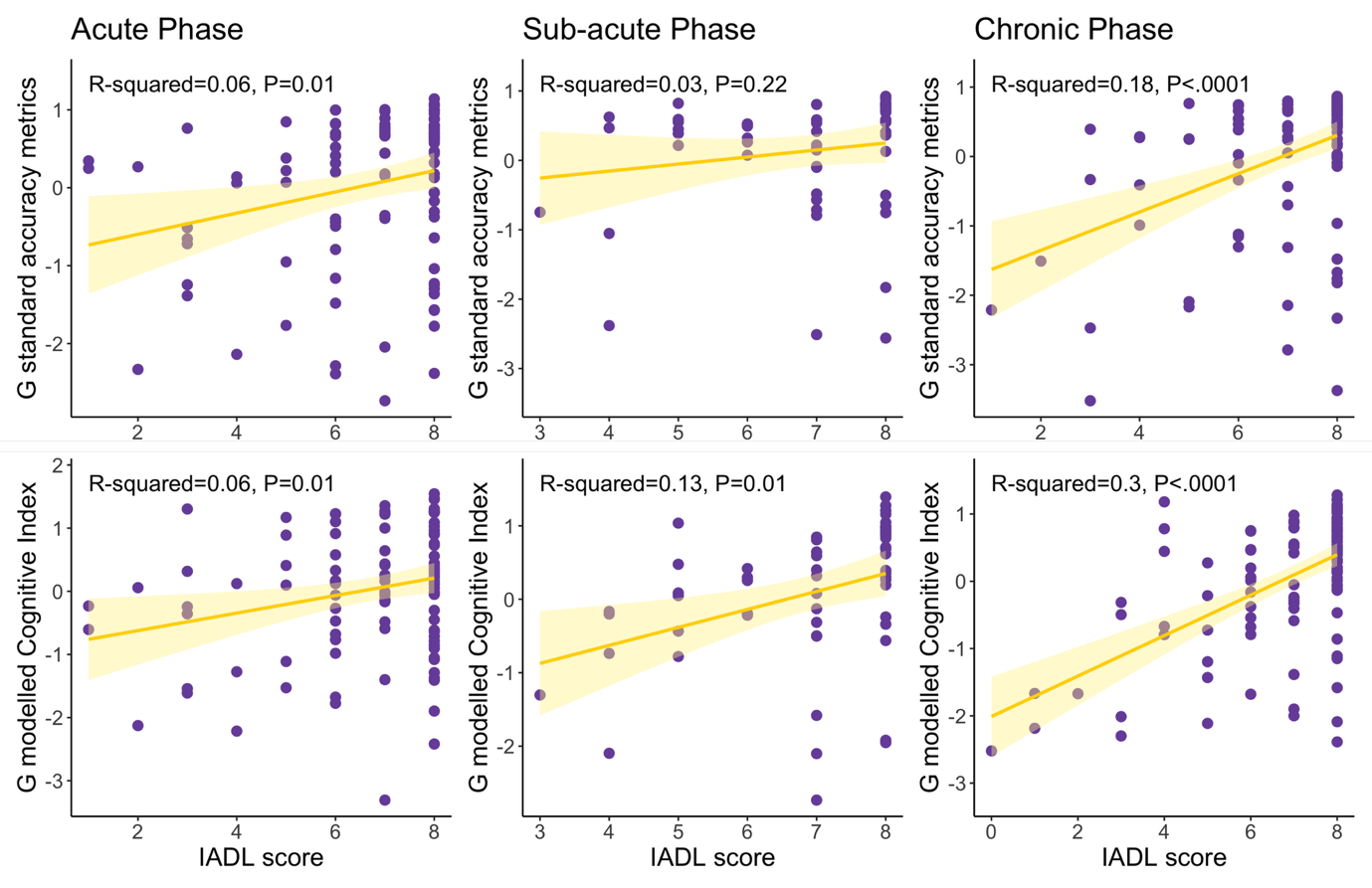


**Supplementary Figure 7.** Lesion overlap map in stroke survivors. Panel A indicates the overlap of the stroke lesion volume, while Panel B indicates the overlap for white matter hyperintensities. A higher overlap of lesion volume across patients is represented in green.

**Supplementary Table 1.** Corrected and uncorrected p-values for the for effect of hand motor impairment on cognitive outcomes (see Figure 3). P-value correction is performed via Benjamini-Hochberg FDR.

| **Uncorrected p value** | **Corrected p value** | **Metric** | **Task** |
| --- | --- | --- | --- |
| 0.50 | 0.50 | Accuracy | Orientation |
| 0.20 | 0.26 | AS_scaled | Orientation |
| 0.001 | 0.002 | MedianRT | Orientation |
| 0.005 | 0.01 | DT_scaled | Orientation |
| 0.48 | 0.99 | Accuracy | Task Recall |
| 0.99 | 0.99 | AS_scaled | Task Recall |
| 0.67 | 0.99 | MedianRT | Task Recall |
| 0.85 | 0.99 | DT_scaled | Task Recall |
| 0.04 | 0.10 | Accuracy | PAL |
| 0.05 | 0.10 | AS_scaled | PAL |
| 0.72 | 0.72 | MedianRT | PAL |
| 0.51 | 0.67 | DT_scaled | PAL |
| 0.77 | 0.77 | Accuracy | Digit Span |
| 0.16 | 0.21 | AS_scaled | Digit Span |
| 0.05 | 0.10 | MedianRT | Digit Span |
| 0.03 | 0.10 | DT_scaled | Digit Span |
| 0.40 | 0.54 | Accuracy | Spatial Span |
| 0.60 | 0.60 | AS_scaled | Spatial Span |
| 0.37 | 0.54 | MedianRT | Spatial Span |
| 0.31 | 0.54 | DT_scaled | Spatial Span |
| 0.005 | 0.02 | Accuracy | Language Comprehension |
| 0.71 | 0.82 | AS_scaled | Language Comprehension |
| 0.68 | 0.82 | MedianRT | Language Comprehension |
| 0.82 | 0.82 | DT_scaled | Language Comprehension |
| <.0001 | <.0001 | Accuracy | Semantic Judgement |
| 0.46 | 0.46 | AS_scaled | Semantic Judgement |
| 0.20 | 0.27 | MedianRT | Semantic Judgement |
| 0.20 | 0.27 | DT_scaled | Semantic Judgement |
| 0.02 | 0.09 | Accuracy | Blocks |
| 0.31 | 0.62 | AS_scaled | Blocks |
| 0.78 | 0.95 | MedianRT | Blocks |
| 0.95 | 0.95 | DT_scaled | Blocks |
| <.0001 | <.0001 | Accuracy | Trail making |
| 0.49 | 0.66 | AS_scaled | Trail making |
| 0.24 | 0.48 | MedianRT | Trail making |
| 0.75 | 0.75 | DT_scaled | Trail making |
| 0.81 | 0.98 | Accuracy | Odd One Out |
| 0.98 | 0.98 | AS_scaled | Odd One Out |
| 0.09 | 0.35 | MedianRT | Odd One Out |
| 0.18 | 0.35 | DT_scaled | Odd One Out |
| 0.51 | 0.58 | Accuracy | Rule Learning |
| 0.58 | 0.58 | AS_scaled | Rule Learning |
| 0.18 | 0.37 | MedianRT | Rule Learning |
| 0.07 | 0.27 | DT_scaled | Rule Learning |
| 0.07 | 0.13 | Accuracy | Pear Cancellation |
| 0.03 | 0.10 | AS_scaled | Pear Cancellation |
| 0.23 | 0.23 | MedianRT | Pear Cancellation |
| 0.10 | 0.13 | DT_scaled | Pear Cancellation |
| <.0001 | <.0001 | Accuracy | SRT |
| 0.06 | 0.11 | AS_scaled | SRT |
| 0.08 | 0.11 | MedianRT | SRT |
| 0.11 | 0.11 | DT_scaled | SRT |
| <.0001 | <.0001 | Accuracy | Auditory Attention |
| 0.05 | 0.10 | AS_scaled | Auditory Attention |
| 0.6 | 0.8 | MedianRT | Auditory Attention |
| 0.8 | 0.8 | DT_scaled | Auditory Attention |
| <.0001 | <.0001 | Accuracy | CRT |
| 0.57 | 0.85 | AS_scaled | CRT |
| 0.83 | 0.85 | MedianRT | CRT |
| 0.85 | 0.85 | DT_scaled | CRT |
| <.0001 | <.0001 | Accuracy | Motor Control |
| 0.001 | 0.003 | AS_scaled | Motor Control |
| 0.90 | 0.90 | MedianRT | Motor Control |
| 0.71 | 0.90 | DT_scaled | Motor Control |
| 0.003 | 0.01 | Accuracy | Calculation |
| 0.09 | 0.09 | AS_scaled | Calculation |
| 0.04 | 0.05 | MedianRT | Calculation |
| 0.03 | 0.05 | DT_scaled | Calculation |
| 0.01 | 0.04 | Accuracy | Gesture Recognition |
| 0.06 | 0.10 | AS_scaled | Gesture Recognition |
| 0.09 | 0.10 | MedianRT | Gesture Recognition |
| 0.10 | 0.10 | DT_scaled | Gesture Recognition |

**Supplementary Table 2.** Corrected and uncorrected p-values for the for the correlations between global cognitive metrics and brain imaging metrics (see Figure 5). P-value correction is performed via Benjamini-Hochberg FDR.

| **Uncorrected p value** | **Corrected p value** | **Metric** | **MRI modality** |
| --- | --- | --- | --- |
| 0.12 | 0.14 | Accuracy | Sub-acute stroke lesion |
| 0.04 | 0.06 | Accuracy | Sub-acute WMH lesion |
| 0.10 | 0.13 | Accuracy | Chronic stroke lesion |
| 0.01 | 0.03 | Accuracy | Chronic WMH lesion |
| 0.02 | 0.04 | Cognitive Index | Sub-acute stroke lesion |
| 0.005 | 0.02 | Cognitive Index | Sub-acute WMH lesion |
| 0.58 | 0.58 | Cognitive Index | Chronic stroke lesion |
| 0.002 | 0.02 | Cognitive Index | Chronic WMH lesion |
